# Detecting early egocentric and allocentric impairments deficits in Alzheimer’s disease: an experimental study with virtual reality

**DOI:** 10.3389/fnagi.2015.00088

**Published:** 2015-05-20

**Authors:** Silvia Serino, Francesca Morganti, Fabio Di Stefano, Giuseppe Riva

**Affiliations:** ^1^Applied Technology for Neuro-Psychology Lab, Istituto Auxologico ItalianoMilan, Italy; ^2^Department of Human and Social Sciences, University of BergamoBergamo, Italy; ^3^Ospedale Castelli VerbaniaVerbania, Italy; ^4^Department of Psychology, Università Cattolica del Sacro CuoreMilan, Italy

**Keywords:** virtual reality, egocentric representation, allocentric representation, mild cognitive impairment, Alzheimer’s disease

## Abstract

Several studies have pointed out that egocentric and allocentric spatial impairments are one of the earliest manifestations of Alzheimer’s Disease (AD). It is less clear how a break in the continuous interaction between these two representations may be a crucial marker to detect patients who are at risk to develop dementia. The main objective of this study is to compare the performances of participants suffering from amnestic mild cognitive impairment (aMCI group), patients with AD (AD group) and a control group (CG), using a virtual reality (VR)-based procedure for assessing the abilities in encoding, storing and syncing different spatial representations. In the first task, participants were required to indicate on a real map the position of the object they had memorized, while in the second task they were invited to retrieve its position from an empty version of the same virtual room, starting from a different position. The entire procedure was repeated across three different trials, depending on the object location in the encoding phase. Our finding showed that aMCI patients performed significantly more poorly in the third trial of the first task, showing a deficit in the ability to encode and store an allocentric viewpoint independent representation. On the other hand, AD patients performed significantly more poorly when compared to the CG in the second task, indicating a specific impairment in storing an allocentric viewpoint independent representation and then syncing it with the allocentric viewpoint dependent representation. Furthermore, data suggested that these impairments are not a product of generalized cognitive decline or of general decay in spatial abilities, but instead may reflect a selective deficit in the spatial organization Overall, these findings provide an initial insight into the cognitive underpinnings of amnestic impairment in aMCI and AD patient exploiting the potentiality of VR.

## Introduction

Given the rise in life expectancy and concomitant growth of the aging population (aged 65 and over), the prevalence of dementia is expected to increase dramatically. It is estimated that the number of the elderly affected by Alzheimer’s disease (AD), which is the most common type of dementia, will reach 115.4 million by 2050 (Prince et al., 2013). Accordingly, the identification of early indicators of cognitive decline in the elderly is becoming a worldwide health policy priority. In parallel with continuous research of well-validated biomarkers of AD processes, cognitive assessment continues to provide reliable cognitive indicators that are crucial for better definition, for both early and differential diagnosis, for improving the design of clinical trials, and for offering the chance of prevention treatments. The early impairment in episodic memory is traditionally considered the first sign of AD (Weintraub et al., 2012). Episodic memory is the ability to encode, store and then retrieve personal past events characterized by a specific time and place (“what,” “where,” and “when”), and with a reference to the individual themselves as participants of those events (Tulving, 1985, 2001, 2002). Indeed, the core feature of episodic memory is the *autonoetic consciousness*, namely the subjective and conscious experience of mentally reliving an event (Tulving, 2001). From a cognitive standpoint, this first-person perspective is the default mode for information processing, and corresponds to egocentric spatial representations (Vogeley et al., 2004). There are two types of spatial representations, defined on the basis of the reference used to encode and store spatial information: egocentric and allocentric representations (Paillard, 1991; Klatzky, 1998).

Egocentric spatial representations are constituted by subject-to-object spatial relations, since spatial information is acquired and processed using the self as the reference (self-centered). These transient spatial representations are integrated in posterior parietal area 7a (Zipser and Andersen, 1988; Pouget and Sejnowski, 1992; Lester and Dassonville, 2014). On the other hand, allocentric spatial representations are constituted by object-to-object spatial relations, since spatial information is stored using objects and/or environmental features as reference (world-centered). Hippocampal place cells are supposed to be responsible for the long-term storage of the allocentric representations of space (O’Keefe and Dostrovsky, 1971; Ono et al., 1993; Ekstrom et al., 2003).

Starting from the role of the hippocampus in providing a spatial scaffold to bind all neocortical representations related to a specific event (O’Keefe and Nadel, 1978; Nadel and Moscovitch, 1997; Moscovitch and Nadel, 1998), the spatial mechanism underlying episodic retrieval has been modeled in the well-known Boundary Vector Model (Burgess et al., 2001; Byrne et al., 2007). According to this model, a retrieval cue (for example, a particular song associated with a meaningful life episode) may evoke the entire past event: the retrieved content includes the spatial scaffold of this past event (i.e., its spatial context), encoded as allocentric representation in the hippocampal regions (i.e., the distances of event elements, which are independent of the individual). Although allocentric, this hippocampal representation is translated into an egocentric representation (i.e., the distances of event elements to the left or right of or ahead of the individual). In this perspective, the difficulty in encoding and storing egocentric and allocentric spatial representations may become a useful cognitive marker of AD. A recent systematic review of allocentric and egocentric abilities in AD showed that there is a prevalence of allocentric deficit both in amnestic mild cognitive impairment (aMCI) and AD patients (Serino et al., 2014). In addition, two selected studies pointed out a more specific cognitive impairment in the translation between the egocentric and allocentric representations (Morganti et al., 2013; Pai and Yang, 2013). These findings underlined that, from the earliest stages of AD, there is a significant degeneration centered in the hippocampus and interconnected areas. Indeed, earliest AD-related neuropathologic changes (i.e., neurofibrillary tangles and amyloid plaques) usually begin in the medial temporal lobe and related areas, especially the hippocampus (Braak and Braak, 1991, 1996; Dickson, 1997; Thal et al., 2000; Alafuzoff et al., 2008). On the other hand, from a cognitive point of view, this review observed a more complex spatial deficit involving the ability to encode and store an allocentric hippocampal representation and, then, to translate it to the egocentric parietal representation. To explain the presence of both allocentric and translation impairments from the earliest stages of AD, Serino and Riva (2013) proposed that early damage in the hippocampus may provoke a break in the mental frame syncing between different spatial representations and, then impair both spatial and episodic retrieval. Indeed, Behrendt (2013) recently proposed a distinction between two types of allocentric representations: the *allocentric view-point dependent representation*, namely an allocentric representation of the scene toward which the individual orients; and the *allocentric view-point independent representation*, namely a complete abstract object-to-object allocentric representation of the scene. From a neurobiological perspective, within the hippocampus there are two regions responsible for the storing of allocentric information, region CA3 and region CA1 (Robertson et al., 1998; Rolls, 2007). More precisely, the dentate gyrus projects to region CA3, which encodes an allocentric viewpoint dependent representation. Then, region CA3 projects, through the Schaffer collaterals, to region CA1, which stores an allocentric viewpoint independent representations. In this perspective, the mental frame syncing may be defined as the ability in the synchronization between these two allocentric spatial representations that is useful for an effective retrieval (Serino and Riva, 2013; Serino et al., 2014). Indeed, when we retrieve an experienced environment and/or a past event, first, we have to encode and memorize an abstract structure of the spatial scene, including all of the relevant objects and their reciprocal relationships (allocentric viewpoint-independent representation). Second, we have to impose a specific viewpoint on this abstract allocentric scene (allocentric viewpoint-dependent representation), to ease its translation into a first-perspective egocentric representation. When there is a break in this process, as it is assumed to happen in AD, the retrieved content may lack coherence.

Virtual reality (VR) appears to be a useful tool to detect early impairment in the ability to encode, store and sync different spatial representations. Besides the opportunity for controlled and secure testing environments (for a review, see Bohil et al., 2011), with VR it is possible to systematically change the retrieval viewpoint with respect to the view-point in the encoding phase. This strategy, known as “virtual disorientation,” induces interference in the egocentric representation and forces the use of long-term allocentric representation (Bosco et al., 2008).

Based on these premises, the main objective of this study is to explore the cognitive underpinnings of spatial impairments in AD using a VR-based procedure specifically designed for evaluating the abilities to encode, use and sync different spatial representations. To achieve this general aim, we will compare the performances of elderly participants suffering from aMCI, patients with AD, and a control group (CG), using both a traditional standard neuropsychological assessment of spatial functions and this VR-based procedure. First, we assumed, in line with the available literature, that both aMCI patients and AD patients will show severe spatial deficits.

Second, based on the “mental frame syncing hypothesis,” we assumed that there are differences between the three groups in our VR-based procedure. Specifically, we argued that AD patients would show a break in the syncing between different spatial representations.

## Materials and Methods

### Participants

A total of 45 participants allocated to three groups were included in the study: 15 AD patients (AD group), 15 aMCI patients (aMCI group), and 15 cognitively healthy individuals (CG). Demographic and clinical characteristics are reported in **Table 1**.

**Table 1:** Demographic and clinical information.

Group			
Test or variable	aMCI group^1^	AD group^1^	CG^1^
Male Female Total	11 4 15	11 4 15	9 6 15
Years of age^2^	77.53 (5.52)	82.93 (5.61)	73.87 (7.38)
Years of education^2^	7.73 (4.48)	6.60 (3.83)	12.27 (3.88)
Duration of disease (months)^2^	–	25.62 (10.45)	–
MMSE^2,3^	22.46 (1.95)	23.06 (1.50)	27.52 (1.48)

^1^amnestic mild cognitive impairment group (aMCI group); Alzheimer’s Disease group (AD group); control group (CG).

^2^Values are shown as mean (SD).

^3^Mini Mental State Examination (MMSE; Folstein et al., 1975).

Individuals for AD group were recruited from the clinically diagnosed outpatients of the Ospedale Castelli Verbania in Verbania (Italy). These diagnoses were made by the clinical geriatric staff using the criteria of the National Institute of Neurological Disorders and Stroke and the Alzheimer’s Disease and Related Disorders Association (McKhann et al., 1984).

Individuals for the aMCI group were recruited from different social senior centers located in Lombardy (Italy). They met the criteria for an amnestic single-domain form of MCI as defined by Petersen (2004), including the presence of a subjective memory complaint; the objective evidence of memory impairment [as assessed by the Short Story Recall call (Spinnler and Tognoni, 1987)]; preserved general cognitive function as assessed by mini mental state examination (MMSE; Folstein et al., 1975); preserved activities of daily living as reported by formal and/or informal caregiver, and the absence of dementia [as assessed by the Milan Overall Dementia Rating Scale (MODA; Brazzelli et al., 1994)]. To verify if the aMCI patients were impaired only on declarative memory functioning, together with their MMSE scores (individuals in this group were required to have a MMSE score > 24, indicating no severe cognitive impairment), we administered the Milan Overall Dementia Rating Scale [MODA, (Brazzelli et al., 1994)] to exclude the presence of dementia and significant impairments in other cognitive domains. Only patients with a score > 63/100, which corresponds to a mild degree of cognitive impairment, and with a performance resulting <1.5 standard deviations below normative norms on the Short Story Recall (Spinnler and Tognoni, 1987), were included in this study.

The CG was recruited from a panel of volunteers. They were eligible to take part in the study if they were over 65 years of age and had no history of traumatic brain injury or any other neurological illness, that may affect brain structures. Individuals in this group were required to have a MMSE score > 27.

Participants did not receive money as reward for the participation to the study and gave their written content for the inclusion in the study, which was approved by the Ethical Committee of Università Cattolica del Sacro Cuore di Milano.

### Traditional Spatial Neuropsychological Assessment

To evaluate the spatial abilities of the study’s participants, the following standard neuropsychological tests were administered. Scores obtained from these neuropsychological tests were corrected for age, education level, and gender according to Italian normative data where needed.

#### Corsi Block Test

The Corsi Block Test (Corsi, 1972; Spinnler and Tognoni, 1987) was used to measure short-term spatial memory (Corsi Span) and long-term spatial memory (Corsi Supraspan). Stimuli consisted of a random array of wooden blocks spread out on a wooden base, placed between the experimenter and the participant. In the Corsi Span, the participants are invited to tap a sequence of wooden blocks in the same order as the researcher, with increasing span length on each trial. In the Corsi Supraspan, the researcher proposed a sequence of nine blocks to be repeated for several trials.

#### Money Road Map

The Money Road Map is used to evaluate spatial navigation abilities (Money et al., 1965). In this test, the participants were given a map of a small town on which was drawn a route taken by a traveler. The route had 32 turns with left–right intersections. The participants had to imagine themselves traveling along this route to decide whether a right or left turn was demanded at the intersections. No time limit was imposed and the maximum score is 32 points.

#### Manikin’s Test

The Manikin’s Test (Ratcliff, 1979) was used to evaluate general mental rotation abilities. The participants were given 32 sheets showing a “little man” from different perspectives who holds a ball. Participants were required to evaluate in which hand the little man was holding the ball. No time limit was imposed and the maximum score is 32 points.

#### The Judgment of Line Orientation

The Judgment of Line Orientation (Benton et al., 1978) was used to assess visuo-spatial skills. Participants were given 30 sheets showing pairs of target lines positioned above a reference figure containing 11 lines arranged in a semicircle and numbered from 1 to 11. They were required to identify their angular positions in relation to the reference figure. No time limit was imposed and the maximum score is 30 points.

### Apparatus and Stimuli

**FIGURE 1:**
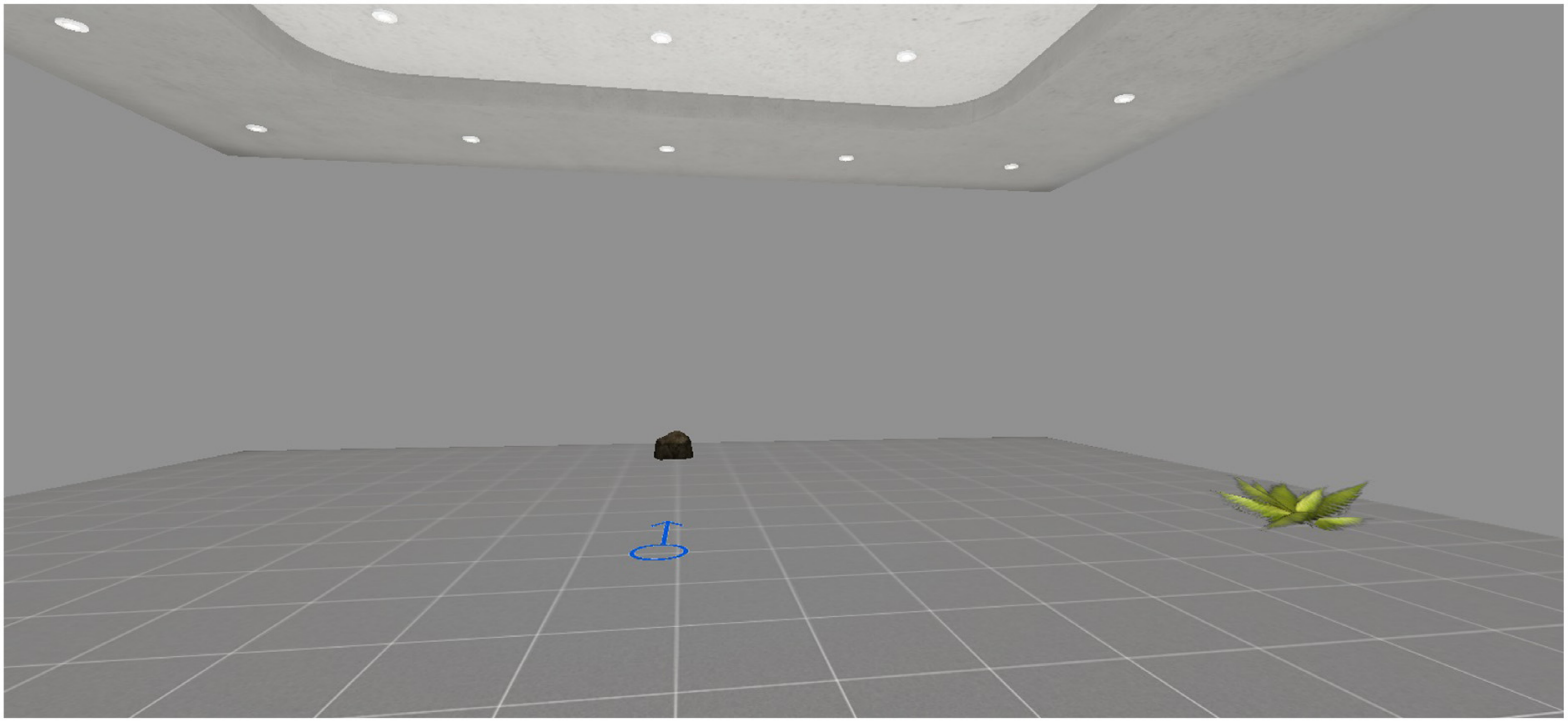
**In the encoding phase, participants were asked to memorize the position of the plant starting from the arrow**.

A virtual room was created as test environment. It included two objects (namely, a plant and a stone) and an arrow drawn on the floor, which pointed to the North and represented the start of the navigation (see **Figure 1**).

As explained later in the procedure, the participants were instructed to memorize the position of the plant, that varied across three different trials. In the first trial (Trial 1), the object in the learning phase was on the East side, in the second trial (Trial 2) the object was on the West side, in the third trial (Trial 3) the object was on the South side. For the retrieval phase, two different tasks were developed. In the first task (Task 1), participants were asked to indicate the position of the object on a real map, namely, a retrieval with spatial allocentric information independent of point of view. In the second task (Task 2), participants were asked to enter an empty version of the same virtual room. The participants had to indicate the position of the plant, starting from the position of the other object, namely, a retrieval without any spatial allocentric information. In this task, the participants changed their points of view from those they had in the learning phase. As posited by Bosco et al. (2008), this strategy induced interference in the egocentric representation of the object with respect to participants’ view (i.e., “virtual disorientation”). To indicate the position of the plant, this technique forced the participants to refer to their allocentric viewpoint-independent representation and sync it with the allocentric viewpoint-dependent representation. In both tasks, the accuracy of spatial location is the dependent variable [0 = no answer; 1 = very poor answer, for example, choosing the same side of the retrieval, namely the North; 2 = poor answer, for example, choosing the opposite side of the virtual room (i.e., choosing the southern side when the object in the learning phase was in the northern part); 3 = medium answer, for example bad left–right discrimination (i.e., the eastern part of the virtual room, when the object in the learning phase was in the western side); 4 = correct answer)].

From a technical point of view, this VR-based procedure for assessing the abilities to encode, use and sync different spatial representations was created using NeuroVirtual 3D, a recent extension of the software NeuroVR (Riva et al., 2011; Cipresso et al., 2014), which is a free VR platform for creating virtual environments, useful for neuropsychological assessment and neurorehabilitation.

### Procedure

Before starting the experimental procedure, each participant was provided with written information about the study and was asked to sign the informed consent form to participate in the study. Then, all participants were required to complete the neuropsychological tests described above. At the start of the experimental session, the participants were seated in front of a horizontally placed 15^′′^ monitor. The monitor screen was placed at a distance of 50 cm from the body plane. The virtual environments were rendered using a portable computer (ACER ASPIRE with CPU Intel® Core^TM^i5 with graphic processor NVidia GeForce GT 540M, 1024 × 768 resolution). The participants also had a gamepad (Logitech Rumble F510), which allowed them to explore and to interact with the environment. After an initial training in VR technology, the experimental procedure was initiated, consisting of an encoding phase, which was followed by the retrieval phase in two different tasks, i.e., Task 1 and Task 2. In the encoding phase, starting from the center of the virtual room (i.e., indicated by the presence of the arrow), each participant was instructed to memorize the position of the plant. In Task 1, the participants were given a real map and invited to retrieve the position of the plant they had memorized in the learning phase, and sign that position with a pen. This real map was a full aerial view of the virtual room. In Task 2, the participants entered the virtual room from the position of the other object (i.e., the stone), and were invited to retrieve the position of the plant they had discovered in the learning phase. They were instructed to stop when they were sure that they had the correct position (i.e., where the plant had been). The order of the presentation of the conditions was randomized for each participant. As explained before, in the Trial 1, the plan was on the East side, in the Trial 2 the object was on the West side, and in the Trial 3 the object was on the South side. The order of the presentation of the trials was randomized for each participant. There was no time limit. Then, all participants were required to indicate the position of the object in the two tasks.

### Data Analysis

First, to investigate differences in the traditional spatial neuropsychological tests, a series of analysis of variance with the LSD *post hoc* comparisons were computed with Group (“aMCI group” vs. “AD group” vs. “CG”) as between variable. Then, differences in the accuracy of the spatial location for Task 1 and Task 2 were calculated using two repeated measure analyses of variance: Trials (“Trial 1” vs. “Trial 2” vs. “Trial 3”) as within factors and Group (“aMCI group” vs. “AD group” vs. “CG”) as between factor. For these analyses that were conducted, the Greenhouse-Geisser test statistic was used when the assumption of sphericity was violated. Pairwise comparisons (with Bonferroni’s adjustment) and simple contrasts were computed to compare significant differences.

In addition, a series of linear multiple regression analyses were carried out to determine whether a combination of traditional neuropsychological tests (MMSE, Money Road Map, Corsi Block Test – Span, Corsi Block Test- Supraspan, Manikin’s Test, The Judgment of Line Orientation) were associated with performance on the VR-procedure.

For all analyses, determination of significance was based on α = 0.05.

## Results and Discussion

Data were entered into Microsoft Excel and analyzed using SPSS version 18 (Statistical Package for the Social Sciences–SPSS for Windows, Chicago, IL, USA).

One patient from the aMCI group was excluded from the analyses of the Corsi Block Test -Supraspan and the Judgment of Line Orientation due to unfinished tasks.

A series of analysis of variance with the LSD *post hoc* comparisons were computed with Group (“aMCI group” vs. “AD group” vs. “CG”) as between variable to investigate differences in the traditional spatial neuropsychological tests. In regard to the Corsi Block Test – Span, findings showed significant differences between groups [*F*(2,42) = 5.174, *p* < 0.05, $\eta_{p}^{2}$ = 0.198]. Post-hoc comparisons showed that the AD group had significantly poor short-term spatial mnestic abilities (*M* = 4.38, SD = 0.83) when compared with the CG (*M* = 5.167, SD = 0.62, *p* < 0.01). As concerns the Corsi Block Test – Supraspan, results indicated significant differences between groups [*F*(2,41) = 13.138, *p* < 0.001, $\eta_{p}^{2}$ = 0.391]. Post-hoc comparisons showed that the CG had better long-term mnestic spatial abilities (*M* = 13.33, SD = 5.86) both when compared with the aMCI group (*M* = 7.96, SD = 3.77 *p* < 0.01) and AD group (*M* = 5.54, SD = 2.32 *p* < 0.001).

Regarding the Money Road Map, results showed significant differences between groups [*F*(2,43) = 3.48, *p* < 0.05, $\eta_{p}^{2}$ = 0.142]. Specifically, *post hoc* comparisons showed that the AD group showed weak spatial navigation abilities (*M* = 16.73, SD = 5.60) when compared with the CG (*M* = 7.96, SD = 3.77, *p* < 0.05).

In relation to Manikin’s Test, findings indicated significant differences between groups [*F*(2,42) = 23.42, *p* < 0.001, $\eta_{p}^{2}$ = 0.527]. Specifically, *post hoc* comparisons showed that the AD group had weak mental rotation abilities (*M* = 17.07, SD = 3.05) when compared with the CG (*M* = 28.73, SD = 3.55, *p* < 0.001). Moreover, it was noted that the AD group performed significantly more poorly compared to the aMCI group (*M* = 23.13, SD = 6.63, *p* < 0.01).

Finally, as concerns the Judgment of Line Orientation, findings showed significant differences between groups [*F*(2,41) = 26.79, *p* < 0.001, $\eta_{p}^{2}$ = 0.567]. *Post hoc* comparisons showed that the AD group had very poor visuo-spatial abilities (*M* = 6.33, SD = 5.46) both when compared with the aMCI group (*M* = 16.57, SD = 7.77 *p* < 0.01) and CG (*M* = 21.73, SD = 3.82 *p* < 0.001). Moreover, mean scores of the aMCI group were significantly higher (*p* < 0.001) when compared with those of the AD group.

On one side, these data indicated that AD patients had severe deficits on all spatial functions analyzed. On the other side, it was noted that the performance of aMCI group is similar to AD patients for almost all the traditional neuropsychological tests considered. Specifically, AD patient are more impaired in mental rotation abilities and in visuo-spatial functions when compared to aMCI group. **Table 2** summarizes findings from spatial neuropsychological tests.

**Table 2:** Analysis of variance results of mean scores obtained by participants divided into the three groups at the spatial neuropsychological tests.

	Group					*Post hoc* comparisons^3^			
	aMCI group^1^	AD group^1^	CG^1^	*F*	*P^3^*	$\eta_{p}^{2}$	aMCI group^1^ vs. AD group^1^	aMCI group^1^ vs. CG^1^	CG^1^ vs. AD group^1^
Corsi Block Test- Span^2^	4.73 (0.50)	4.38 (0.83)	5.16 (0.62)	5.17	^∗∗^	0.198	N.S.	N.S	^∗∗^
Corsi Block Test- Supraspan^2^	7.96 (3.77)	5.54 (2.32)	13.33 (5.86)	13.13	^∗∗∗^	0.391	N.S.	^∗∗^	^∗∗∗^
Money Rood Map^2^	16.73 (5.63)	16.80 (3.09)	21.20 (6.59)	3.48	^∗^	0.142	N.S.	N.S.	^∗∗^
Manikin’s Test^2^	23.13 (6.63)	17.07 (3.05)	28.73 (3.55)	23.42	^∗∗∗^	0.527	^∗∗^	^∗∗^	^∗∗∗^
Judgment of Line Orientation^2^	16.57 (7.77)	6.33 (5.46)	21.73 (3.82)	26.79	^∗∗∗^	0.567	^∗^	^∗∗∗^	^∗∗∗^

^1^amnestic mild cognitive impairment group (aMCI group); Alzheimer’s Disease group (AD group); control group (CG).

^2^Values are shown as mean (SD).

^3∗^*p* < 0.05, ^∗∗^*p* < 0.01, ^∗∗∗^*p* < 0.001; N.S., non-significant.

As concerns data from the VR-based procedure, two repeated measure analyses of variance were carried out. One patient from the aMCI group was excluded from the analyses due to unfinished tasks.

First of all, to investigate differences in the accuracy of the spatial location for Task 1, a repeated measure analysis of variance was computed: Trials (“Trial 1” vs. “Trial 2” vs. “Trial 3”) as within factors and Group (“aMCI group” vs. “AD group” vs. “CG”) as between factor. No significant effect of Group was found, i.e., there were no absolute significant differences between groups in the ability to retrieve spatial allocentric information independent of point of view. The main effects of Trial [*F*(1,82) = 18.09, *p* ≤ 0.001, $\eta_{p}^{2}$ = 0.306] were significant. Specifically, simple contrasts indicated that the average scores were significantly lower in the third trials, when compared to the first trial [*F*(1,41) = 17.73, *p* < 0.001, $\eta_{p}^{2}$ = 0.302], and to the second trial [*F*(1,41) = 27.02, *p* < 0.001, $\eta_{p}^{2}$ = 0.394]. The third trial may be more difficult since the object is presented at the South of the virtual room in the encoding phase, requiring a 180° spatial rotation to find it. Finally, a significant effect was found of the interaction Trials X Group [*F*(4,82) = 4.40, *p* < 0.01, $\eta_{p}^{2}$ = 0.177]. As shown by simple contrasts, aMCI patients performed significantly more poorly in the third trial when compared to CG [*F*(2,41) = 4.81, *p* < 0.01, $\eta_{p}^{2}$ = 0.190] and to AD group [*F*(2,41) = 5.03, *p* < 0.01, $\eta_{p}^{2}$ = 0.197].

Second, to investigate differences in the accuracy of the spatial location for Task 2, another repeated measure analysis of variance was conducted: Trials (“Trial 1” vs. “Trial 2” vs. “Trial 3”) as within factors and Group (“aMCI group” vs. “AD group” vs. “CG”) as between factor.

The main effect of Group was found [*F*(2,41) = 2.41, *p* < 0.05, $\eta_{p}^{2}$ = 0.161]. Specifically, *post hoc* comparisons indicated that AD patients performed more poorly (*M* = 2.71, SD = 1.57) when compared with the CG (*M* = 3.33, SD = 1.57, *p* < 0.05). This means that AD patients showed very weak abilities in retrieving the position of the object without allocentric spatial information. Moreover, results indicated significant differences within Trials [*F*(2,1,517) = 8.48, *p* < 0.01, $\eta_{p}^{2}$ = 0.177]. As for the Task 1, the Trial 3 appeared to be the most difficult. Specifically, simple contrasts indicated that the average scores were significantly lower in the Trial 3 when compared to the Trial 1 [*F*(1,41) = 19.37, *p* < 0.01, $\eta_{p}^{2}$ = 0.321] and to the Trial 2 [*F*(1,41) = 6.16, *p* < 0.01, $\eta_{p}^{2}$ = 0.131]. No significant interaction effect Trials X Group was found, i.e., all groups performed worse in the Trial 3.

**Table 3** summarizes mean scores obtained by participants in both tasks.

**Table 3:** Mean scores obtained by participants divided into the three groups at the virtual-reality based procedure for evaluating abilities in encoding, using and syncing spatial representations.

	Task	Trial	Mean	SD
aMCI group^1^	Task 1	First trial	3.89	0.01
	Task 1	Second trial	3.86	0.36
	Task 1	Third trial	2.57	0.93
	Task 2	First trial	3.13	0.51
	Task 2	Second trial	2.93	0.79
	Task 2	Third trial	2.07	0.96
AD group^1^	Task 1	First trial	3.53	0.73
	Task 1	Second trial	3.87	0.51
	Task 1	Third trial	2.93	1.03
	Task 2	First trial	3.13	0.51
	Task 2	Second trial	2.93	0.79
	Task 2	Third trial	2.07	0.96
CG^1^	Task 1	First trial	3.73	1.03
	Task 1	Second trial	3.73	0.45
	Task 1	Third trial	3.60	0.82
	Task 2	First trial	3.33	1.11
	Task 2	Second trial	3.60	0.50
	Task 2	Third trial	3.07	1.43

^1^amnestic mild cognitive impairment group (aMCI group); Alzheimer’s Disease group (AD group); control group (CG).

Finally, a series of linear multiple regression analyses, including all participants, with the accuracy of spatial location for both tasks in each trials as the dependent variable, and general cognitive functioning (MMSE) and traditional Money Road Map, Corsi Block Test – Span, Corsi Block Test- Supraspan, Manikin’s Test, The Judgment of Line Orientation) as independent variables, were carried out. All independent variables were entered singularly into the model using the ‘enter’ method. Results revealed that these neuropsychological tests in combination with each other did not predict impairment in the ability to retrieve spatial allocentric information independent of point of view, not in the Trial 22 (*R*^2^ = 0.141, *p* = 0.561), nor in the Trial 2 (*R*^2^ = 104, *p* = 0.743), nor in the Trial 3 (*R*^2^ = 0.305, *p* = 0.71).

As concerns findings from the second tasks, results showed that these neuropsychological tests in combination with each other predict impairment in the ability to retrieve the position of the object without allocentric spatial information only in the Trial 2 (*R*^2^ = 0.375, *p* < 0.05) and in the Trial 3 (*R*^2^ = 0.381, *p* < 0.05), but not in the Trial 1 (*R*^2^ = 0.279, *p* = 0.743). However, findings revealed that there are only two significant predictors of performance in the third trial of the Task 2, namely, the scores on the Money Road Map (*B* = -0.389, *t* = -2.140, *p* < 0.05) and the scores on the Manikin’s Test (*B* = -0.687, *t* = 2.774, *p* < 0.01). These two tests, indeed, evaluate respectively the ability in the spatial navigation, which requires the cognitive ability to correctly retrieve the position of the object in large environment, and the mental rotation ability, which is fundamental in the Trial 3, since it required a 180° spatial rotation to memorize the object.

Together, these data suggested that the impairments in the encoding, using and syncing between different spatial representations are not a product of generalized cognitive decline (as measured with the MMSE) or of general decay in spatial abilities, but instead may reflect a selective deficit in spatial organization.

## Conclusion

It is well known that spatial memory deficits characterize the cognitive profile of AD patients (Iachini et al., 2009; Gazova et al., 2012; Lithfous et al., 2013). These spatial impairments manifest themselves in several episodes of topographical disorientation, which were reported in both AD outpatients (McShane et al., 1998) and AD patients residing in a community (Pai and Jacobs, 2004). What is still under debate in scientific literature are the cognitive underpinnings of spatial memory deficits in AD, and the relationship with early impairment in the episodic memory.

If spatial memory can be defined as the ability to encode and store information from our surrounding in egocentric and allocentric representations (O’Keefe and Nadel, 1978), how can deficits in the relationships between these representations become a crucial early indicators of cognitive decline? Within this research field, the current study is aimed at comparing the performances of elderly participants suffering from amnestic MCI, AD patients and a CG, using a VR-based procedure for assessing the ability to encode, use and sync different spatial representations.

First, in line with previous research and clinical evidence mentioned, our results confirmed that AD patients were impaired in the traditional neuropsychological evaluation of spatial functions when compared with the CG. Specifically, it was observed that the cognitive profile of aMCI group is very similar to AD patients for almost all the spatial traditional neuropsychological tests considered. Since the introduction of the clinical criteria in the late 1990s (Petersen et al., 1999), the concept of MCI has been used both in clinical and in research settings to identify individuals in the early stages of cognitive impairment. In particular, amnestic MCI patients are more likely to develop AD when compared with cognitively healthy age-matched individuals (Mitchell and Shiri-Feshki, 2009).

As concerns results from the VR-based procedure, our findings showed that in both tasks all groups performed more poorly in the Trial 3 (i.e., the plant is at the southern side of the virtual room during the encoding phase), which may be more difficult since it required a 180° spatial rotation to memorize the object. On one side, our finding showed that aMCI patients, compared with cognitively healthy controls and AD patients, performed significantly more poorly in the Trial 3 of Task 1. In the Trial 3 of the task, aMCI patients showed a specific deficit in the ability to encode and store an allocentric viewpoint independent representation, since this task asked participants to retrieve the position of the object on a real map. On the other side, our findings from Task 2 indicated that AD patients, compared with cognitively healthy controls, had a specific impairment in syncing the allocentric viewpoint independent representation with the allocentric viewpoint dependent representation. As previously explained, Task 2 may evaluate a more complex spatial ability since participants are required to indicate the position of the object in an empty virtual room without any spatial allocentric information, starting from another point of view. Thus, this task forced the participants to refer to their stored allocentric viewpoint-independent representation and sync it with the allocentric viewpoint-dependent representation. Finally, our results suggested that the impairments in the encoding, using and syncing between different allocentric representations are not a product of generalized cognitive decline (as assessed with the MMSE) or of general decay in spatial abilities, but instead may reflect selective deficits in the spatial organization.

In sum, according to the “mental frame syncing” hypothesis (Serino and Riva, 2013, 2014), our data indicated the presence of a deficit in storing an allocentric viewpoint independent representation in aMCI patients. Then, a profound deficit was found in AD patients in the storage of an allocentric viewpoint independent representation and, consequently, in its synchronization with the allocentric viewpoint dependent representation. From a neurobiological perspective, Padurariu et al. (2012) have recently showed that decrease of hippocampal neuronal density in AD is more prominent, especially in the CA1 and CA3 hippocampal areas. As previously explained, these early neurodegenerative processes significantly impair the neural network that is presumed to be crucial for storing and syncing allocentric representations. The synchronization between the allocentric viewpoint independent representation and the viewpoint dependent representation permits a coherent spatial framework, which is crucial for an effective spatial and episodic retrieval (Serino and Riva, 2014). Moreover, on the basis of the most recent theories of episodic memory, several cognitive and neural processes work in parallel to support the aforementioned “mental time travel” from past to present and future (for a review, see Roediger et al., 2007). Specifically, a number of studies have shown that when individuals remember the past or imagine the future, a comparable level of activation occurs in the medial temporal and frontal lobes, the posterior cingulate, the retrosplenial cortex, and the lateral parietal area (Okuda et al., 2003; Addis et al., 2007, 2009; Buckner and Carroll, 2007; Botzung et al., 2008; Spreng et al., 2009; Viard et al., 2011; Eichenbaum, 2013). Within a wider theoretical account, Buckner and Carroll (2007) theorized that the so-called default network (which includes the above mentioned area of activation) serves as “self-projection,” with the ability to shift perspective from the immediate present to alternative perspectives. In addition to the default network’s role in remembering the past and imagining the future (i.e., episodic memory) and simulating another viewpoint for successfully orienting in space (i.e., spatial memory and navigation), this includes the ability to conceive the viewpoint of others [i.e., “theory of mind” (TOM)]. An interesting systematic review showed that recent evidence underlined the existence of impairment in the most complex TOM tasks in AD, but it is still unclear whether a TOM deficit is linked to global cognitive dysfunctions or to a specific dysfunction in the episodic memory system (Moreau et al., 2013). According to Frith and de Vignemont (2005), in the egocentric viewpoint, the others are represented in relation to the self, while in the allocentric perspective, the others’ mental states are represented independently from the self. However, there is no empirical evidence of the underlying cognitive mechanism that supports this process, and what happens if there is an impairment. It would be interesting, as a future challenge, to investigate if a deficiency in the storage of an allocentric viewpoint independent representation and, then, in its syncing with the allocentric viewpoint dependent representation, which affects the possibility to create a coherent scaffold for an effective retrieval of our experiences, may also explain the difficulty in the cognitive translocation of our current viewpoint in other viewpoints.

The findings of this study are interesting and valuable, but there are some limitations. First, one limitation of our study is the difference between the patients and CGs in terms of age and years of educations. Scores obtained from spatial neuropsychological battery were corrected for age and education level according to Italian normative data where needed, but the findings from VR-based procedure must be viewed according to this potential limit. Second, in relation to the use of virtual tools the neuropsychological evaluation of cognitive function, it would also be useful to assess the patient’s perception of usability (for example, difficulties during the experience in using the joystick). However, it is interesting to note that only one patient from the aMCI group did not complete the task. Third, it would be crucial to carry out a longitudinal study to investigate the progression from a deficit in storing allocentric viewpoint independent representation deficit to a more subtle impairment in the synchronization between different allocentric representations across time in the same sample of patients.

In conclusion, although preliminary, these findings provide an initial insight on the cognitive underpinnings of mnestic impairment in aMCI and AD patients. A more precise evaluation of cognitive abilities exploiting the potentiality of VR would offer also the chance to detect subtle deficits in early stages of AD.

## Conflict of Interest Statement

The authors declare that the research was conducted in the absence of any commercial or financial relationships that could be construed as a potential conflict of interest.
